# COVID-19 in Chronic-Phase Chronic Myeloid Leukemia Patients: A Single-Center Survey from Turkey

**DOI:** 10.4274/tjh.galenos.2020.2020.0472

**Published:** 2021-02-25

**Authors:** Umut Yılmaz, Aslıhan Pekmezci, Yalçın Gül, Ahmet Emre Eşkazan

**Affiliations:** 1 İstanbul University-Cerrahpaşa, Cerrahpaşa Faculty of Medicine, Department of Internal Medicine, Division of Hematology, İstanbul, Turkey; 2 İstanbul University-Cerrahpaşa, Cerrahpaşa Faculty of Medicine, Department of Internal Medicine, İstanbul, Turkey

**Keywords:** Chronic myeloid leukemia, COVID-19, Drug-drug interaction, QTc prolongation, SARS-CoV-2, Tyrosine kinase inhibitor

## To the Editor,

The management of patients with hematological malignancies including chronic myeloid leukemia (CML) can be challenging in the era of the coronavirus disease-19 (COVID-19) pandemic [1]. There are limited data published in the literature reporting the outcomes of CML patients with COVID-19 [2,3], so, herein, we share our experiences with the management of patients with CML with severe acute respiratory syndrome-coronavirus-2 (SARS-CoV-2) infection.

The Ministry of Health of the Republic of Turkey has been recording every test for SARS-CoV-2 in the public health records, which are accessible to physicians. We identified 243 adult chronic-phase CML patients currently receiving medical care from our institution and acquired their public health records for SARS-CoV-2 polymerase chain reaction analysis from nasopharyngeal swab specimens. Sixteen of our patients had undergone testing for SARS-CoV-2 by 7 July 2020, and five patients (5/243; 2%) tested positive. Table 1 shows selected features of these infected patients. The total number of documented COVID-19 infections in Turkey at that time was approximately 207,000 (0.25% of Turkey’s population). All five patients received a diagnosis of CML in the chronic phase and currently have optimal responses under tyrosine kinase inhibitor (TKI) treatment. Three patients were continuing imatinib treatment since their diagnosis. Two patients were receiving nilotinib, one due to imatinib failure and the other due to imatinib intolerance. The only notable TKI toxicity with the current treatments was QTc prolongation observed in Patient #4 in 2013 under nilotinib, which required a dose reduction. All patients underwent thorax computed tomography (CT), which showed features of pneumonia in three cases. All five patients recovered without the need for oxygen support, and TKI treatment was interrupted in the two patients who used nilotinib due to concerns of drug-drug interactions and QTc prolongation. The other three patients were receiving imatinib, which was continued during the infection in two patients and withheld in one. Four patients received prophylactic enoxaparin (Patients #1, #3, #4, and #5), and three received hydroxychloroquine and macrolide combinations (Patients #1, #2, and #4). Patient #3 received favipiravir. Only one patient was administered an antibacterial other than a macrolide (Patient #1), and only one patient (Patient #5) received anti-influenza medication.

The eleven patients who tested negative for COVID-19 were contacted by telephone. Five of them had been asymptomatic and were tested due to contacts. Two patients were tested during the evaluation of symptoms of the upper respiratory tract, from which they recovered without a complicated course. One patient was tested while being evaluated for diarrhea and another while being prepared for a cystoscopy. Two patients were tested before travel. All of these eleven patients were in good health when they were interviewed.

CML can present unique problems during a SARS-CoV-2 infection [1]. Drug-drug interactions between TKIs and COVID-19 treatments can be hazardous and require careful monitoring. The chronic side effects of TKIs, including myelosuppression, fluid retention, pulmonary toxicity (dasatinib) [4], and increased risk of thrombosis (nilotinib, ponatinib) [5], tolerated in an otherwise healthy state, may become intolerable during SARS-CoV-2 infection. Disease-specific factors of CML that influence the course of COVID-19 also await identification.

Three (60%) of our five patients had lung involvement according to a chest CT scan. This rate seems to be high. However, two of these patients underwent chest CT only because they were assumed to be in a high-risk group due to CML and had no signs or symptoms related to the lungs. Thus, comparing the rate of lung involvement in our CML cohort with the rate of pneumonia in the general population may be misleading as the imaging indications are biased and the number of infected patients in our cohort is small.

The papers by Li et al. [2] and Breccia et al. [3] report a total of 17 CML patients who contracted COVID-19, and the prevalence in their respective cohorts was 0.9% and 0.17%. Three of these 17 patients died during SARS-CoV-2 infection. We found a prevalence of 2% with no fatalities in our cohort. These values are difficult to compare as the numbers are low and the methods and the timings are different. TKIs are proposed to be protective against COVID-19 [3]; however, some consequences of continuing TKIs during COVID-19 also raise concerns. When our data are included, seven patients (five imatinib [2], one dasatinib [6], one flumatinib [2]) continued TKI treatment during COVID-19 with one fatality. Four patients who discontinued TKIs recovered uneventfully [2]. Another recent paper from the Netherlands found no alarming events in a CML cohort of 148 patients regarding the COVID-19 pandemic [7]. Thus, the decision to withhold or continue TKIs during COVID-19 is difficult and requires careful consideration of multiple factors, including the patient’s co-morbidities, co-medications, TKIs, CML response status, previous adverse events, COVID-19 treatments, and the severity of the infection.

In conclusion, we report five CML patients with COVID-19, all of whom recovered without the need for intensive care. CML patients may have disease-related or treatment-related factors that place them at a higher risk of complications during SARS-CoV-2 infection. A diligent and individualized approach is necessary. Documentation of this growing experience is necessary to optimize the care for these patients.

## Figures and Tables

**Table 1:**
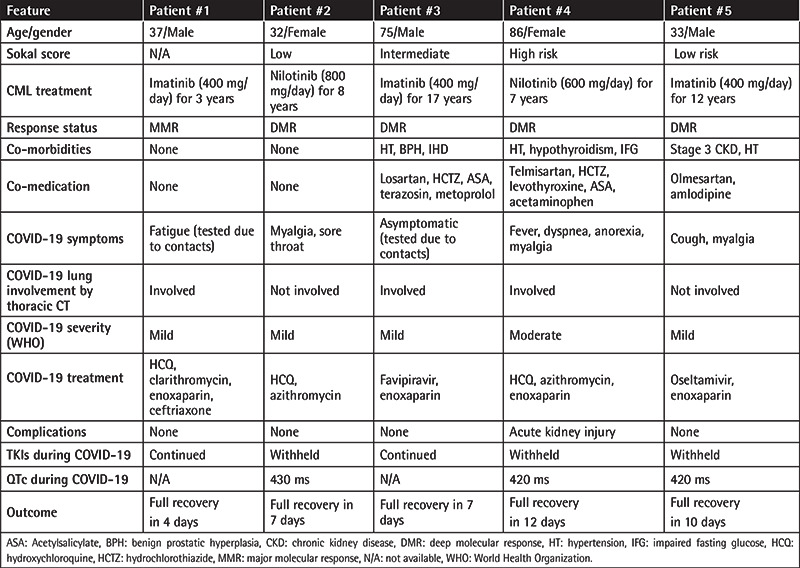
Clinical features of the CML-CP patients that contracted SARS-CoV-2.

## References

[1] Eşkazan AE (2020). Chronic myeloid leukaemia and the use of tyrosine kinase inhibitors in the days of COVID-19 pandemic. Br J Clin Pharmacol.

[2] Li W, Wang D, Guo J, Yuan G, Yang Z, Gale RP, You Y, Chen Z, Chen S, Wan C, Zhu X, Chang W, Sheng L, Cheng H, Zhang Y, Li Q, Qin J; Hubei Anti-Cancer Association, Meng L, Jiang Q (2020). COVID-19 in persons with chronic myeloid leukaemia. Leukemia.

[3] Breccia M, Abruzzese E, Bocchia M, Bonifacio M, Castagnetti F, Fava C, Galimberti S, Gozzini A, Gugliotta G, Iurlo A, Latagliata R, Luciano L, Pregno P, Rege-Cambrin G, Rosti G, Stagno F, Tiribelli M, Foà R, Saglio G;, Campus CML Working Group (2020). Chronic myeloid leukemia management at the time of the COVID-19 pandemic in Italy. A campus CML survey. Leukemia.

[4] Eskazan AE, Soysal T, Ongoren S, Gulturk E, Ferhanoglu B, Aydin Y (2011). Pleural and pericardial effusions in chronic myeloid leukemia patients receiving low-dose dasatinib therapy. Haematologica.

[5] Hochhaus A, Baccarani M, Silver RT, Schiffer C, Apperley JF, Cervantes F, Clark RE, Cortes JE, Deininger MW, Guilhot F, Hjorth-Hansen H, Hughes TP, Janssen JJWM, Kantarjian HM, Kim DW, Larson RA, Lipton JH, Mahon FX, Mayer J, Nicolini F, Niederwieser D, Pane F, Radich JP, Rea D, Richter J, Rosti G, Rousselot P, Saglio G, Saußele S, Soverini S, Steegmann JL, Turkina A, Zaritskey A, Hehlmann R (2020). European LeukemiaNet 2020 recommendations for treating chronic myeloid leukemia. Leukemia.

[6] Abruzzese E, Luciano L, D’Agostino F, Trawinska MM, Pane F, De Fabritiis P (2020). SARS-CoV-2 (COVID-19) and chronic myeloid leukemia (CML): A case report and review of ABL kinase involvement in viral infection. Mediterr J Hematol Infect Dis.

[7] Ector GICG, Huijskens EGW, Blijlevens NMA, Westerweel PE (2020). Prevalence of COVID-19 diagnosis in Dutch CML patients during the 2020 SARS-CoV2 pandemic. A prospective cohort study. Leukemia.

